# Prevalence of Vancomycin-Resistant Enterococci colonization and its risk factors in chronic hemodialysis patients in Shiraz, Iran

**DOI:** 10.1186/1471-2334-7-52

**Published:** 2007-06-06

**Authors:** Ojan Assadian, Mehrdad Askarian, Maria Stadler, Soheila Shaghaghian

**Affiliations:** 1 Department of Hygiene and Medical Microbiology, Medical University Vienna, Vienna General Hospital, Vienna, Austria; 2 Department of Community Medicine, Shiraz University of Medical Sciences, Shiraz, Ira; 3 Shiraz Nephro-urology Research Center, Shiraz, Iran

## Abstract

**Background:**

Vancomycin-resistant entrococci (VRE) are increasing in prevalence at many institutions, and are often reported in dialysis patients. The aim of this cross-sectional prevalence study was to determine the prevalence and risk factors of VRE colonization in chronic hemodialysis patients in two hemodialysis centers in Shiraz, Iran.

**Methods:**

Rectal swabs were obtained from all consenting patients and were streaked on the surface of Cephalexin-aztreonam-arabinose agar (CAA) and incubated at 37°C in air for 24 h. The vancomycin susceptibility of each isolate was confirmed by disk susceptibility testing. The MICs of vancomycin and teicoplanin were confirmed by the E test. To identify risk factors, a questionnaire was completed for all the studied patients and the data of VRE positive and negative groups were compared using Man-Withney U test for continues data and the Fisher exact test for categorical data.

**Results:**

Of 146 patients investigated, 9 (6.2%) were positive for VRE. All VRE strains were genotypically distinguishable. Risk factors for a VRE-positive culture were "antimicrobial receipt within 2 months before culture" (P = 0.003) and "hospitalization during previous year" (P = 0.016).

**Conclusion:**

VRE colonization is an under-recognized problem among chronic dialysis patients in Iran. VRE colonization is associated with antibiotic consumption and hospitalization.

## Background

The emergence and spread of glycopeptide resistance in enterococci has become a substantial clinical and epidemiological concern, and vancomycin-resistant enterococci (VRE) are now an increasingly important problem in hospitals worldwide [1]. In 1999, a Greek study conducted to determine the increased spread of VRE colonization reported a prevalence of VRE colonization of 1.2%. In 2003, however, the corresponding figure was 34.9% [2]. Increase of VRE poses several challenges, including firstly the sole availability of expensive new antimicrobials for therapy of infections due to VRE, since most VRE are also resistance to multiple other economically acceptable drugs in developing countries, e.g., aminoglycosides or ampicilin, and secondly the possibility that the vancomycin resistance genes present in VRE may be transferred to other gram-positive microorganisms such as *Staphylococcus aureus *[3].

Patients with chronic renal failure undergoing hemodialysis are at increased risk for acquiring VRE [1]. In an U.S. study, 17.8% of hemodyalysis patients became colonized with VRE, an incidence rate of one case per 9.8 patient-years of follow-up [4].

In the so far only existing – but unpublished – Iranian study, the risk of VRE colonization in hemodialysis patients was 3.78 times higher than that of all other patients [5]. Previous studies showed significant association between VRE colonization in hemodialysis patients and several factors including non-ambulatory status [6,7], hospitalization [6,8,9], intensive care unite (ICU) stays [8], receipt of antibiotic [6] use of vancomycin [4,6-8,10], age [11], type of dialysis [9], anemia [9], and leukocytosis [9]. Yet, because the reasons for the higher prevalence in hemodialysis patients are not fully clear and because there is only limited data for Iran, we decided to study risk factors for VRE colonization in these patients.

## Methods

This cross-sectional prevalence study was conducted in the two referral dialysis center of Shiraz. (Namazee and Faghihi Hospital). Shiraz is the largest city in the southern part of Iran. The university hospitals in Shiraz serve as referral centers for one-quarter of the Iran's medical cases with about 21,000 admissions per year. Rectal swabs were obtained from all consenting hemodialysis patients (n = 146) of these dialysis centers during March 2005. Acute hemodialysis patients were excluded from this cross-sectional study. These swabs were immediately transported to the microbiology laboratory using cephalexin-aztrenam-arabinose agar transport medium.

### Culture media and condition

Cephalexin-aztreonam-arabinose agar (CAA) was prepared by adding 40 g of Columbia agar base (Unipath, Basingstoke, United Kingdom), 10 g of arabinose (Sigma Chemical Co., Poole, United Kingdom), and 3.6 mL of phenol red (2%; BDH, Lutterworth, United Kingdom) to 1 liter of de-ionized water. The medium was mixed, and the pH was adjusted to 7.8. The agar was autoclaved at 114°C for 20 minutes. Fresh sterile solutions of aztreonam (Bristol-Myers Squibb, Hounslow, United Kingdom) and cephalexin (Sigma) were added to give final concentrations of 75 and 50 mg/liter, respectively [12]. Swabs were streaked on the surface of CAA agar. The plates were incubated at 37°C in air for 24 h and examined for growth and fermentation of arabinose. A change in the color of the medium surrounding the colony, from red to yellow, indicated arabinose fermentation.

### Conventional organism identification and susceptibility testing

Red to yellow colonies resembling enterococci on the CCA plates were further identified by conventional laboratory methods, including Gram staining and determination of colonial morphology and biochemical and growth characteristics [13]. Isolates that were likely VRE were screened for vancomycin resistance by using brain heart infusion agar (BHIA) containing 6 mg of vancomycin per liter and BHIA containing 16 mg of vancomycin per liter. The vancomycin susceptibility of each isolate was confirmed by disk susceptibility testing by National Committee for Clinical Laboratory Standards (NCCLS) methods [14]. The MICs of vancomycin and teicoplanin for the isolates were further determined by the Etest (AB Biodisk).

### Molecular typing

Molecular typing of VRE strains was performed using random-amplified polymorphic DNA (RAPD) according to the method described by Barbier et al. earlier [15]. Primer P15 was used. PCR products were run on 1.5% agarose gels and stained with ethidium bromide.

### Data acquisition and statistical analysis

Study personnel used standardized forms to abstract data from the patient's medical records. A baseline form was completed on all patients, which contained data on demographic information, cause of end-stage renal disease, duration of renal replacement therapy, frequency of dialysis, antibiotic consumption during previous 2 month, hospitalization during previous year, mobility status and albumin level. We selected these items for evaluation about risk factors of VRE colonization after a review of items was used in similar studies. The data were entered into and analyzed with SPSS version 11.5. Standard descriptive statistical techniques were used to determine prevalence of VRE colonization among hemodialysis patients. For determining risk factors for VRE colonization, the VRE positive and negative groups were compared using Man-Whitney U test for continuous and the Fisher exact test for categorical data. For all analyses, a type 1 error of 0.05 was applied.

The study was approved by the local ethics committee. Ethical considerations including privacy of personal data were regarded during all of the steps of the research. The questionnaires did not include any personal identification.

## Results

A total of 146 patients (79 male) with a median age of 50 (range: 18–81) years were screened for VRE. The median duration of dialysis treatment was 18 months (range: 1 – 168). Most patients had diabetes mellitus (29.5%) and hypertension (43.8%) as an underlying disease, and 23.3% of them had limited motility function. Only 1.4% of the studied patients had received antibiotic during the previous 2 months, and 6.8% hospitalized during the previous year. (Table 1)

**Table 1:** Based-line characteristics and comparison of hemodialysis patients cultured versus not cultured for VRE colonization- Shiraz; 2005

	Total	VRE	No VRE	P value
	
- Patients	146	9	137	-
- age (years), median (range)	50 (18–81)	50 (18 – 81)	57 (24 – 72)	0.389
- Gender, male: female	67:79	5:4	74:63	1.000
- Underlying kidney disease				1.000
- Diabetes mellitus	43 (29.5%)	3 (33.3%)	40 (29.2%)	
- Hypertension	64 (43.8%)	4 (44.4%)	60 (43.8%)	
- Others	39 (26.7%)	2 (22.2%)	37 (27%)	
- Duration of dialysis (months), median (range)	18 (1–168)	18 (1 – 168)	15.5 (6 – 64)	0.758
- Frequency of dialysis, median (range) per month	12 (4–12)	12 (4 – 12)	8 (8 – 12)	0.486
- Serum albumin concentration (mg(dl), median (range)	4.2 (2.6–5.4)	4.2 (2.6 – 5.4)	4.3 (3.5 – 5)	0.572
- Motility status				0.213
- Normal	112 (76.7%)	5 (55.6%)	107 (78.1%)	
- Limited	34 (23.3%)	4 (44.4%)	30 (21.9%)	
- Antibiotic consumption during previous 2 month	2 (1.4%)	2 (22.2%)	0 (0%)	0.003
- Hospitalization during previous year	10 (6.8%)	3 (33.3%)	7 (5.1%)	0.016

**Figure 1:**
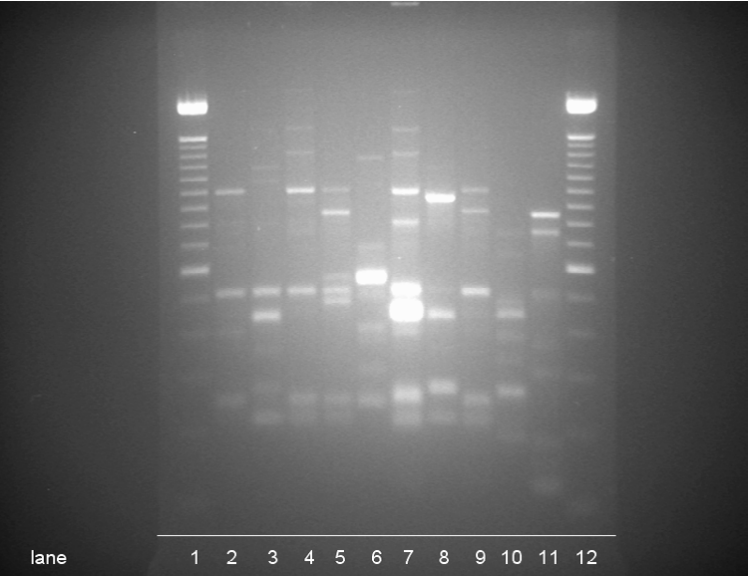
RAPD-PCR typing of 9 VRE isolates including 1 not related control strain (lane 11). Legend: Lane 1 and 12: 100 bp lambda ladder; lanes 2 – 10: VRE strains isolated from chronic dialysis patients in Shiraz, Iran; lane 11: not related VRE strain isolated at the Vienna General Hospital in March 2007.

Among these patients, 9 (6.2%) were colonized with VRE. All VRE strains were genotypically distinguishable (Figure 1). None of the patients presented with infection. Various factors were analyzed as possible predictors of VRE colonization including: age, sex, underlying kidney disease, duration of dialysis, frequency of dialysis, serum albumin concentration and motility status (Table 1). The only statistically significant differences between VRE-positive and VRE-negative patients were "antibiotic consumption during previous 2 month" (P = 0.003) and "hospitalization during previous year" (P = 0.0016). Both patients having received antibiotics within the previous 2 months also have been hospitalized during the past year.

## Discussion

Patients with chronic renal failure who are undergoing hemodialysis are at increased risk for acquiring VRE. Hemodialysis-dependent patients are at increased risk for VRE for several reasons: they have extensive contact with healthcare system, they are often in close proximity to other VRE patients, they frequently have multiple co-morbid conditions, and they often receive repeated prolonged courses of antibiotics including vancomycin [1].

To our knowledge, this study is the first to define the prevalence of VRE colonization in a cohort of long-term outpatient dialysis patients in Iran. We found VRE rectal carriage in 6.2% of hemodialysis patients, a result similar to those reported in a number of previous studies that have examined VRE colonization among dialysis patients. VRE prevalence was 9.5% at the center affiliated with the university of Maryland hospital [16], 9% among 111 dialysis patients near New York City [10], 6% at the Vanderbilt University Medical Center [7], and 8.1% at Johns Hopkins University Hospital [4].

An increased risk of VRE infection and colonization has been associated with non-ambulatory status [6,7], receipt of antibiotic [6], hospitalization [6,8,9], ICU stays [8], use of vancomycin [4,6-8,10], anemia [9], and leukocytosis [9]. However, our study found only association between hospitalization and antibiotic consumption with VRE colonization. Interestingly, both patients having received antibiotics within the previous 2 months also have been hospitalized during the past year. The factor 'hospitalization during the past year' might well be a surrogate for antibiotic consumption. It can be speculated that the small number of VRE isolate limited our ability to evaluate risk factors. Furthermore, the yield of rectal swabs when compared to fresh stool samples has been demonstrated not to be high [17], and the use of broth enrichment might have increased the yield in VRE. Novicki et al. demonstrated in 2004 [18] that addition of a broth enrichment step leads to the detection of significantly more VRE isolates than direct plating alone. Therefore, the used sampling technique might be a second potential limitation of our study with regards to underestimating the true prevalence as well as potential misclassification of controls.

Yet, Reisner et al. [19] clearly showed that use of an enrichment broth medium is required to recover VRE contaminating environmental surfaces; however, direct inoculation to selective solid medium is adequate to recover VRE in patient perianal specimens. The purpose of our study, however, was not to determine a precise estimation of the prevalence in hemodialysis patients, but to obtain enough information to calculate risk factors for VRE carriage. Furthermore, in an epidemiological sense pertaining to the risk of transmission it remains debatable how relevant a VRE carrier is, if the isolate is only detectable by means of enrichment cultures.

Because there is no commonly agreed regimen to eradicate VRE colonization, efforts to preventing VRE's spread are paramount [8]. Current experience emphasizes continued enforcement of infection-control measures and prudent use of antibiotics. These interventions may have a beneficial impact on the rapidly rising rates of VRE among chronic hemodialysis patients. However, improving compliance with infection control measures and prudent use of antibiotics will be challenging, since generally, compliance is low.

## Conclusion

VRE colonization is an under-recognized problem among chronic dialysis patients in Iran. VRE colonization is associated with antibiotic consumption and hospitalization.

## Abbreviations

BHIA Brain heart infusion agar

VRE Vancomycin-resistant Enterococci

ICU Intensive Care Unite

CAA Cephalexin-aztreonam-arabinose

NCCLS National Committee for Clinical Laboratory Standards

## Competing interests

The authors have no financial or other conflict of interest to declare in relation to this manuscript and declare no financial or other relationships leading to a conflict of interest.

## Authors' contributions

Ojan Assadian Conception and design of the study, supervising and coordinating activities in Vienna, analysis and interpretation of data, statistical analysis, drafting and revising the manuscript

Soheila Shaghaghian Acquisition of data, Statistical analysis, revising the manuscript

Maria Stadler Advised on and performed the laboratory work, drafting the manuscript

Mehrdad Askarian Conception and design of the study, supervising and coordinating activities in Shiraz, analysis and interpretation of data, statistical analysis, drafting and revising the manuscript critically for important intellectual content

All authors have read and approved to the final version of the manuscript.

## Pre-publication history

The pre-publication history for this paper can be accessed here:


